# On the inflammatory response in metal-on-metal implants

**DOI:** 10.1186/1479-5876-12-74

**Published:** 2014-03-21

**Authors:** Ulrike Dapunt, Thomas Giese, Felix Lasitschka, Jörn Reinders, Burkhard Lehner, Jan Philippe Kretzer, Volker Ewerbeck, Gertrud Maria Hänsch

**Affiliations:** 1 Department of Orthopaedics and Trauma Surgery, University Hospital Heidelberg, Schlierbacher Landstrasse 200a, 69118 Heidelberg, Germany; 2 Department of Immunology, Heidelberg University, Im Neuenheimer Feld 305, 69120 Heidelberg, Germany; 3 Department of Pathology, Heidelberg University, Im Neuenheimer Feld 224, 69120 Heidelberg, Germany

**Keywords:** Metal-on-metal implants, Metal wear particles, Innate immune response, T-cell response, Cytokines, Osteolysis

## Abstract

**Background:**

Metal-on-metal implants are a special form of hip endoprostheses that despite many advantages can entail serious complications due to release of wear particles from the implanted material. Metal wear particles presumably activate local host defence mechanisms, which causes a persistent inflammatory response with destruction of bone followed by a loosening of the implant. To better characterize this inflammatory response and to link inflammation to bone degradation, the local generation of proinflammatory and osteoclast-inducing cytokines was analysed, as was systemic T cell activation.

**Methods:**

By quantitative RT-PCR, gene expression of cytokines and markers for T lymphocytes, monocytes/macrophages and osteoclasts, respectively, was analysed in tissue samples obtained intraoperatively during exchange surgery of the loosened implant. Peripheral T cells were characterized by cytofluorometry before surgery and 7 to 10 days thereafter.

**Results:**

At sites of osteolysis, gene expression of cathepsin K, CD14 and CD3 was seen, indicating the generation of osteoclasts, and the presence of monocytes and of T cells, respectively. Also cytokines were highly expressed, including CXCL8, IL-1ß, CXCL2, MRP-14 and CXCL-10. The latter suggest T cell activation, a notion that could be confirmed by detecting a small, though conspicuous population of activated CD4+ cells in the peripheral blood T cells prior to surgery.

**Conclusion:**

Our data support the concept that metallosis is the result of a local inflammatory response, which according to histomorphology and the composition of the cellular infiltrate classifies as an acute phase of a chronic inflammatory disease. The proinflammatory environment, particularly the generation of the osteoclast-inducing cytokines CXCL8 and IL1-ß, promotes bone resorption. Loss of bone results in implant loosening, which then causes the major symptoms of metallosis, pain and reduced range of motion.

## Background

In patients with osteoarthritis, replacement of dysfunctional joints by endoprostheses is the therapy of choice. Particularly total hip replacement is rated as the most successful surgical intervention in the field of orthopaedics (reviewed in [1]). An abundance of implants are available at the surgeons demand. Because of direct contact of the implanted devices with tissues and serum mediator systems, materials are designed to be biologically inert, and to allow the ingrowth of tissue cells, such as osteoblasts and fibroblasts without eliciting adverse reactions from the patients.

Unfortunately, implanted materials can release wear particles which may elicit adverse reactions in patients, apparent as localized inflammation with tissue damage and bone degradation which finally results in loosening of the implant (reviewed in [2]).

**Figure 1:**
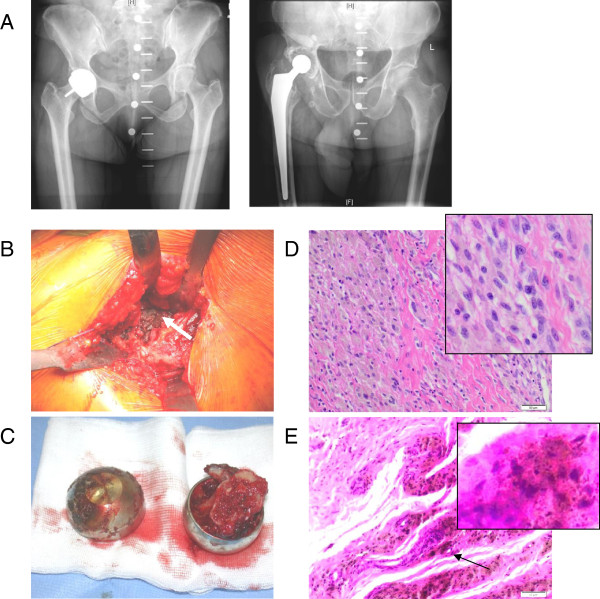
**Clinical and histological images. A** Shown are x-rays of a patient with a metal-on-metal hip resurfacing implant (left) and on the right of a patient with a total hip joint replacement. **B** After the removal of the implant, deposition of metal particles is seen as is the formation of a pseudotumour (arrow). **C** The removed implants are shown; they are "empty" due to bone loss. **D**, **E** Biopsies from an osteolytic site show infiltration of leukocytes, particularly of mononuclear cells, and deposits of metal wear particles.

A special situation arises when so-called metal-on-metal hip resurfacing implants are used. They have been introduced especially for younger, active patients because healthy bone is spared by this device (reviewed in [3,4]) (see also Figure 1). Unfortunately, some metal-on-metal implants release large amounts of wear particles, which are deposited in the tissue and elicit adverse tissue reactions [5-12] (see also Figure 1). This condition has been described many years ago [13-15] and when patients experience severe pain at the site of the implant and reduced range of motion, the implant has to be replaced. So far, specific diagnostic tools are not available, though elevated concentrations of metal ions in the blood point towards an increased wear [16]. Radiologic examination might reveal a deterioration of bone around the implant, which could be responsible for a loosening of the implant. A greyish coloring of the tissue might be seen macroscopically when the implant is removed.

Histology of tissue derived from patients with metallosis shows a wide spectrum of changes. Areas of metal deposits present with granulomatous inflammation, infiltrates of monocytes and T cells, and occasionally giant cells. In some patients tumour-like tissue formations, so-called "pseudotumours", are found in close proximity of the implant, in others a perivascular accumulation of lymphocytes. [17-20]. The difference in morphology could reflect different underlying pathomechanisms, for example distinct responses to a higher versus a lower load of metal particles, or alternatively, different stages of the disease [21,12].

Basically, metallosis is thought to be the result of an immune reaction to the metal wear particles. A participation of phagocytic cells is presumed, because they readily take up particulate materials, and because they are activated during that process to produce pro-inflammatory cytokines (reviewed in [22]). A hypersensitivity towards metals in form of a specific T cell mediated immune response has also been suggested; a classical "type IV immune responses" towards metal/metal ions, however, occurs only in a few patients [23,24], leading to the notion that an allergic reaction to metal ions is not a common denominator of metallosis. Moreover, some studies failed to show typical T cell activation markers or characteristic T cell-derived cytokines [25]. However, due to their short half-life some cytokines might escape detection by conventional methods, and therefore a participation of T cells in the progression of metal-induced tissue damage cannot be excluded. In the present study we examined the local generation of cytokines by gene expression analysis, particularly with regard to T cell-derived cytokines, and the possible link to bone degradation. Moreover, systemic T cell activation in patients was assessed.

## Patients, materials and methods

### Patients

Five patients undergoing revision surgery due to a hip resurfacing implant (metal-on-metal, ASR Hip System, DePuy) and six patients suffering from aseptic loosening of a total hip replacement (metal-on-polyethylene or ceramic-on-polyethylene) were included in the study. Diagnosis of loosening was based on patient’s complaints, clinical examination and by conventional x-ray and/or CT-scan (patients’ clinical data are summarised in Table 1). The study was approved by the local ethic committee, and informed consent was obtained from the patients.

**Table 1:** Patients’ clinical and laboratory findings ( metallosis and aseptic loosening)

**Patient**	**Age**	**Clinical signs**	**Metal ions in peripheral blood**	**CRP (>5 mg/L)**	**White cell count (>10/nl)**	**Duration of implantation (years)**	**Articulating material**
			**a) Chrome (>7 μg/L)**				
			**b) Cobalt (>7 μg/L)**				
**Metallosis**							
1	62	Pain, osteolysis around cup on x-ray	a) 21.3 μg/L	2.3 mg/L	4.55/nl	8	Metal on metal
			b) 37.6 μg/L				
2	47	Pain	a) 17.7 μg/L	16.6 mg/L	8.96/nl	5	Metal on metal
			b) 38.90 μg/L				
3	42	Pain	a) 4.06 μg/L	3.6 mg/L	9.39/nl	6	Metal on metal
			b) 5.49 μg/L				
4	71	Pain	a) 15.0ug/L	6.9 mg/L	5.89/nl	6	Metal on metal
			b) 30.0ug/L				
5	60	Non	a) 43.0 μg/L	2.0 mg/L	5.70/nl	8	Metal on metal
			b) 64.0 μg/L				
**Patient**	**Age**	**Clinical signs**		**CRP (>5 mg/l)**	**White cell count (>10/nl)**	**Duration of implantation (years)**	**Articulating material**
**Aseptic loosening**							
1	70	Pain, osteolysis around cup and stem on x-ray		17.3 mg/l	4.91/nl	19	Ceramic on polyethylene
2	71	Pain, osteolysis around stem on x-ray		10.0 mg/l	8.17/nl	3	Ceramic on polyethylene
3	75	Pain, osteolysis around cup on x-ray		2.0 mg/l	5.74/nl	15	Metal on polyethylene
4	59	Pain, osteolysis around stem on x-ray		2.0 mg/l	3.53/nl	2	Ceramic on polyethylene
5	81	Pain, osteolysis around stem on x-ray		2.0 mg/l	6.92/nl	12	Metal on polyethylene
6	84	Pain, osteolysis around stem on x-ray		2.0 mg/l	6.1/nl	6	Metal on polyethylene

Metal ions were measured pre-operatively as indicator of increased wear debris.

CRP levels and white cell count were measured pre-operatively as signs of infection.

### Collection of tissue and blood samples

During surgery, soft tissue samples were taken from the cup, the capsule, the femur and for comparison from muscle in a standardized fashion. The tissue samples were divided, a part was fixed in formalin and conserved for histological analysis, and another was placed into RNAlater (Ambion, Lifetechnologies, Heidelberg, Germany) for quantitative PCR analysis. Immediately before surgery, and 7 to 10 days thereafter, peripheral blood was collected into heparinized tubes and cells were subjected to cytofluorometry (see below).

### Histology

For routine histological evaluation, the samples were embedded in paraffin, decalcified, and sections of 3 to 4 μM were prepared for eosin-haematoxylin staining.

### Gene expression analysis

The tissue samples were disrupted with RiboLyser devices (ThermoHYBAID, Heidelberg, Germany) containing 400 μl lysis buffer from the MagnaPure mRNA Isolation Kit containing 1%DTT (v/w) (ROCHE Diagnostics, Mannheim, Germany). mRNA was isolated with the MagnaPure-LC device using the mRNA- standard protocol for cells. An aliquot of mRNA was reversely transcribed using AMV-RT and oligo- (dT) as primer (First Strand cDNA synthesis kit, ROCHE Diagnostics, Mannheim, Germany) according to the manufactures protocol in a thermocycler. Primer sets optimized for the LightCycler® (RAS, Mannheim Germany) were purchased from SEARCH-LC GmbH ( http://www.Search-LC.com). The PCR was performed with the LightCycler® FastStart DNA Sybr GreenI kit (RAS) according to the protocol provided. To control for specificity, a melting curve analysis was performed. The copy number was calculated from a standard curve, obtained by plotting known input concentrations of four different plasmids at log dilutions to the PCR-cycle number (CP) at which the detected fluorescence intensity reaches a fixed value. To correct for differences in the content of mRNA, the calculated transcript numbers were normalized according to the expression of the housekeeping gene peptidylprolyl isomerase B (PPIB). Values were thus given as transcripts per 1000 transcripts of PPIB.

### Cytofluorometry

The following antibodies were used: CD4 PerCP, CD8 PerCP, CD11b PE, CD28 FITC (all Becton Dickinson, Heidelberg, Germany), and the respective isotype: mouse IgG1-PerCP, mouse IgG1-PE (all Becton Dickinson, Heidelberg, Germany) and IgG1-FITC (Beckman Coulter, Marseille, France). Whole heparinized blood (100 μl) was incubated with the respective antibodies (20 min, room temperature), erythrocytes were then lysed by Facs Lysing solution and cells were washed and fixed with 1% paraformaldehyde. Cells were analysed by FacsCalibur using CellquestPro 3.0 as software (Becton and Dickinson, Heidelberg, Germany).

### Statistical analysis

Differences between groups were calculated using Friedman test and Mann–Whitney test, respectively (as indicated in the figure legends or tables).

## Results

### Macroscopic and histological aspects of metallosis

Five patients with metallosis and six patients with aseptic loosening were included in the study (patients’ data are summarised in Table 1). The implant used in patients developing metallosis is shown in Figure 1A. In comparison to other forms of endoprostheses, the femur is better preserved, but because of the metal-on-metal contact, metal wear particles accumulate at the site, macroscopically seen as greyish colouring (Figure 1B). In this example, also pseudotumour formation occurred (Figure 1B), as did osteolysis (Figure 1C). Biopsies taken from the site showed metal wear particles, and also a cellular infiltrate, consisting mainly of mononuclear cells (Figure 1D,E).

### Gene expression of cathepsin K, CD3, CD14 and of cytokines in tissue

**Figure 2:**
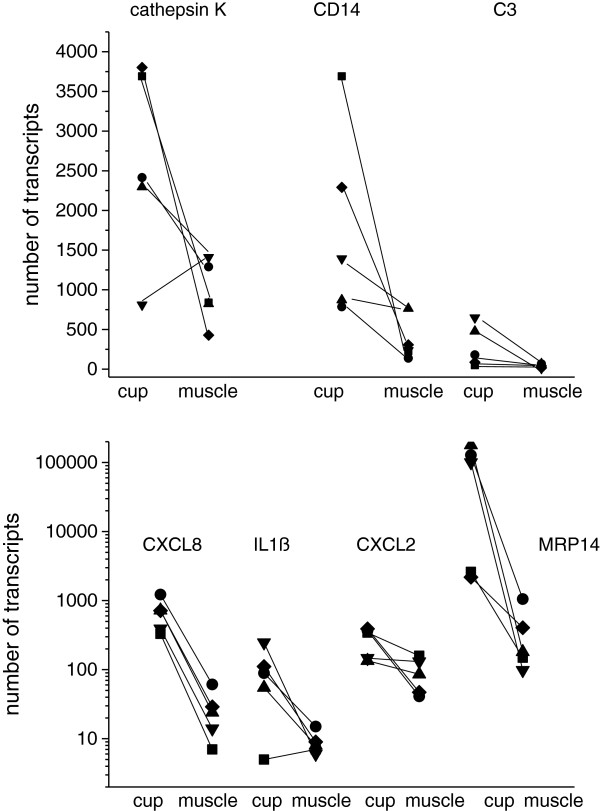
**Expression of cathepsin K, CD14, CD3 and of proinflammtory cytokines in tissue derived from the cup or the muscle: cytokine expression was determined by RT-PCR and quantified as number of transcripts.** Data of 5 patients (each symbol represent one patients) with metallosis are shown.

Tissue samples from the cup and from the femoral bone were taken from areas that were exposed after removal of the implant. Moreover, samples from the capsule and when present from the pseudotumour, were gathered and for comparison, from distant muscle as an unaffected site. Gene expression of CD14 as marker for monocytes/macrophages, of CD3 as marker for T cells and of cathepsin K, characteristic for osteoclasts, was determined by quantitative PCR. At the primary osteolytic site, the cup, expression of cathepsin K, CD14 or CD3 was considerably higher compared to expression in muscle (Figure 2). The absolute numbers varied widely among the patients, but the expression pattern was similar.

In the same tissues, gene expression of proinflammatory cytokines was analysed. CXCL8 (interleukin (IL)-8), IL-1ß, CXCL2 (macrophage inflammatory protein, MIP2α) and MRP14 (S100A9) were highly expressed in tissue of the cup, again with a wide variation among the patients (Table 2). Gene expression of TNFα (tumour necrosis factor alpha), RANK (receptor activator of NfκB) or RANK ligand (RANKL) was rather low in all tissues. The monocyte chemotactic protein, MCP-1 (CCL2) was found in all tissues with no apparent preference, as was CXCL10 (IP-10).

**Table 2:** Gene expression of cathepsin K, CD3, CD14 and of cytokines in tissue of patients with metallosis

**Parameter**	**Cup**	**Muscle**	**Difference between groups calculated by Mann–Whitney test**
Cathepsin K	2603.0 ± 1220.8*	968.4 ± 392.4	n.d.
CD3	288.6 ± 262.2	42.2 ± 26.4	p = 0.036
CD14	1806.2 ± 1211.9	334.2 ± 247.7	p = 0.012
CXCL8	677.0 ± 355.5	27.0 ± 20.8	p = 0.043
IL1ß	101.4 ± 90.7	9.0 ± 3.5	p = 0.043
CXCL2	276.5 ± 125.0	92.8 ± 51.5	p = 0.040
MRP14	82376.8 ± 78340.0	377.4 ± 396.0	p = 0.040
RANK	10.9 ± 9.8	8.2 ± 5.7	n.d.
RANKL	4.1 ± 4.0	0.2 ± 0.2	n.d.
TNFα	11.5 ± 4.0	7.3 ± 1.0	n.d.
CXCL10	184.7 ± 239.5	131.6 ± 147.8	n.d.
CCL2	1612.1 ± 1958.9	652.0 ± 144.8	n.d.

*copy number; n.d. = not different.

To address the question whether aspects of the cytokine expression pattern were typical for metallosis, we compared the data with those obtained from patients requiring replacement of a hip implant because of aseptic loosening.

Because for both patient groups tissue from the capsule and from the femoral bone was available, gene expression in these tissue were used, although these were not the primary osteolytic sites, particularly not in patients with metallosis. As summarised in Table 3, for metallosis patients essentially the same expression pattern was seen: in tissue from either capsule or femoral bone, expression of CXCL8, IL-1ß, CXCL2 and MRP14 was higher compared to muscle tissue, as was expression of CD14. In patients with aseptic loosening, in contrast, no major differences in cytokine expression were observed between muscle and the other tissues; only CD14 was expressed to a higher extent in femoral bone. In patients with metallosis, CXCL8 and IL-1ß expression was higher by trend than in patients with aseptic loosening; CXCL2 and CXCL10 were significantly higher expressed in patients with metallosis (Table 3).

**Table 3:** Cytokine expression in tissue of patients with metallosis versus aseptic loosening

	**Capsule**		**Femoral**		**Muscle**	
	**Metallosis**	**Aseptic loosening**	**Metallosis**	**Aseptic loosening**	**Metallosis**	**Aseptic loosening**
CXCL8	**1927.3 ± 328.4+**	106.2 ± 106.6	**580 ± 316.2**	379.2 ± 494.1	**27 ± 20.8+**	181.3 ± 305.4
IL-1	**179.5 ± 293.5+**	11.2 ± 12.1	**65 ± 44.7**	47.9 ± 48.4	**9 ± 3.5+**	12.2 ± 18.8
RANK	5.6 ± 5.9	9.5 ± 8.8	11 ± 4.5	26.3 ± 32.7	8.2 ± 5.7	19.3 ± 22.9
RANKL	4.4 ± 7	17.3 ± 18.7	11 ± 8.2	44.7 ± 50.2	0.2 ± 0.4	2 ± 4.9
Cathepsin K	2708.8 ± 2139.7	4794.9 ± 2817.2	3313.3 ± 1646.1	6871.6 ± 4937.7	968.4 ± 392.4	2958.5 ± 3390.7
CCL2	2005.7 ± 1321.5	990.7 ± 512.6	3439.8 ± 2892.6	1196.3 ± 575.3	652 ± 144.8	1065.8 ± 1030.5
CD14	**1075.5 ± 659.1+**	**563.2 ± 321+**	823.8 ± 488.9	687.8 ± 417.2	**288.8 ± 151.3 +**	**305.2 ± 175 +**
CD3	64.1 ± 59.8	29.5 ± 15.7	111.5 ± 77.1	62.2 ± 69.9	42.2 ± 26.5	45.7 ± 19.3
TNFα	10.6 ± 10	3.7 ± 2.2	12.8 ± 7	5.7 ± 3.7	5.8 ± 3.3	4.2 ± 6.2
CXCL10	241.4 ± 246.2*	25.9 ± 17.6	183.8 ± 130*	41.7 ± 26.9 *	131.6 ± 147.8	607.2 ± 1167.8
MRP14	4899 ± 2656.3 *	**806 ± 65**	48214.3 ± 91617.3*	**2461.3 ± 2529.1**	377.4 ± 396	2186.7 ± 2080.3
CXCL2	241.2 ± 126.5 *	**73.2 ± 59.2**	670 ± 635.2	133.8 ± 100.2	92.8 ± 51.6	262.3 ± 467.9

The bold print indicates that the groups differ significantly as tested by Mann–Whitney test; * indicates differences between metallosis and aseptic loosening; the + differences between capsule/femoral bone tissue and muscle.

Gene expression of CD3, CD14 and of the cytokines was also determined in peripheral blood cells of the patients. No major differences were observed between blood cells from patients with metallosis or with aseptic loosening (data not shown). Only expression of CXCL10 was higher in blood cells of metallosis patient compared to patients with aseptic loosening (9.4 ± 4.1 copies versus 1.7 ± 1.75).

### Analysis of T cell response

**Figure 3:**
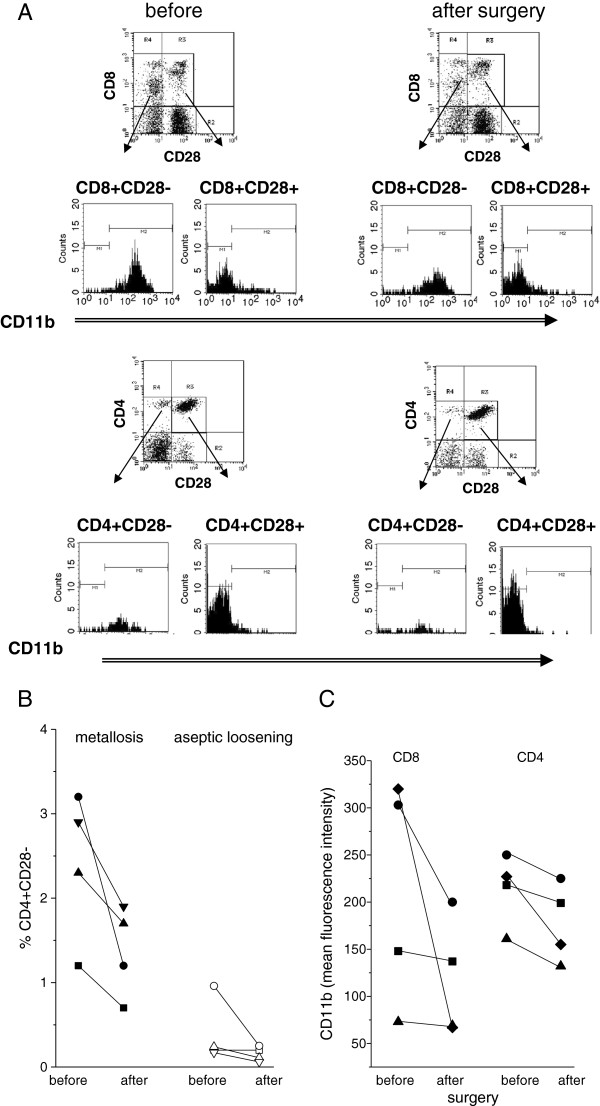
**Cytofluorometry of peripheral T cells. A** By cytofluorometry the peripheral T cells of a patient before and 9 days after surgery were analysed. Before surgery, 57.0% of CD8+ cells were negative for CD28, but only 45.6% after surgery; and 7.4% of CD4 versus 4.6% after surgery. The CD8 + CD28- and CD4 + CD28- cells expressed CD11b, indicative of an activated T effector cell. **B** Shown is the percentage of CD4 + CD28- cells in patients with metallosis (left) or patients with aseptic loosening (right) before and after surgery. **C** The mean fluorescence intensity of CD11b on T cells declined after surgery (shown are data of four patients; each symbol refers to one patient and data obtained before and after surgery are connected by a line).

Peripheral blood T cells were analysed for expression of activation-associated receptors. As sensitive markers, down-modulation of CD28 and up-regulation of CD11b on CD4+ and CD8+ T cells was measured (example in Figure 3A). Because the percentage of CD4 + CD28- and CD8 + CD28- cells varies greatly among donors (mean ± SD of n = 18: 6.51 ± 5.59% CD4 + CD28- and 37.4 ± 20.3% CD8 + CD28-) [26], and because the activation of T cells is transient, we determined the T cell population in the patients immediately before surgery and 7 to 10 days thereafter. There were no major differences regarding CD8 + CD28- cells, whereas CD4 + CD28- cells were seen in patients with metallosis (and not in patients with aseptic loosening) (Figure 3B). CD11b upregulation occurred in both, CD4+ and CD8+, and the percentage declined after surgery, as did the mean values of CD11b (Figure 3C).

## Discussion

To get insights into the local immune response in patients with metal-on-metal implants, tissue derived from osteolytic sites was explored with regard to infiltrating leukocytes, generation of osteoclasts and production of pro-inflammatory cytokines by gene expression analysis. High expression of CD14 and of CD3 indicative of monocyte and T cell infiltration was seen, as was expression of cathepsin K, an enzyme typically expressed by osteoclasts. In osteolytic tissue also genes encoding for pro-inflammatory cytokines, including CXCL8, IL-1ß, CXCL2 and MRP14 were highly expressed, reflecting a localized inflammatory response. Expectedly, the number of gene transcripts varied widely among the patients, reflecting the fact that most likely multiple factors contribute to metallosis, such as the abundance and size of the wear particles; the exposure time of the local cells to wear particles; and also host-inherent factors, particularly the responsiveness of leukocytes to irritants, and their capacity to synthesize and release cytokines.

With regard to osteolysis, gene expression of CXCL8, IL-1ß and CXCL2 are of special interest. These are all multifunctional cytokines that can promote the inflammatory response, for example by attracting and activating more leukocytes. Moreover, CXCL8, IL-1ß and CXCL2 can induce the generation of osteoclasts from precursor cells, either directly or in combination with other cytokines [27-29]. The high expression of these cytokines could explain that osteolysis occurs despite our finding that RANK and RANKL, which are described as crucial in induction of osteoclastogenesis in other conditions, are only marginally expressed [30-32].

In patients with metallosis gene expression of cytokines, particularly of CXCL2 and CXCL10, was higher compared to patients with aseptic loosening of a total joint replacement. Due to the small number of patients and the wide variation among the patients the data have to be interpreted with caution. Possibly, metal wear particles elicit a more prominent inflammation than wear particles derived from metal-on-polyethylene or ceramic-on-polyethylene implants. This explanation is supported by the fact that in the latter implants, expression of most cytokines at osteolytic sites did not exceed that in muscle. A qualitative different inflammatory response, however, cannot be ruled out.

The most likely source for CXCL8, CXCL2, IL-1ß and MRP-14 are phagocytic cells, particularly infiltrated monocytes, which – according to the literature – are activated by wear particles. Monocyte infiltration as determined by CD14 gene expression was higher in patients with metallosis, and to some extent reflects an activation on monocytes, because it is known to be associated with enhanced CD14 synthesis [33]. Whether monocyte activation by metal-on-metal implants differs in principle from metal-on-polyethylene or ceramic-on-polyethylene implants or whether it is only a quantitative difference cannot be decided as yet, nor can we deduce from our data whether this contributes to the lower cytokine expression in patients with aseptic loosening.

CXCL8 and CXCL2 are also chemotactic for T cell subpopulations [34-36] and T cell infiltrates were also seen in patients with metallosis. Infiltration indicates an activation of T cells; as typical T cell-derived cytokine interferon gamma was indirectly assessed by determining CXCL10. In the tissue, expression varied widely among the patients with a trend towards higher expression in patients with metallosis compared to patients with aseptic loosening. CXCL10 was also found in peripheral blood T cells of patients with metallosis, but not in the blood of patients with aseptic loosening. Although the number of transcripts was rather low, the data point to an activation of T cells in metallosis. The interpretation is supported by the presence of CD4+ and CD8+ cells which were CD28 negative and expressed CD11b, a phenotype corresponding to an activated effector cell [26,37]. The percentage of CD4 + CD28- cells declined within days after removal of the implant, as did the percentage of CD4+ or CD8+ cells expressing CD11b. Because CD8 + CD28- are long-lived and remain longer in the circulation, the population of CD8 + CD28- did not change as convincingly as CD4 + CD28-.

The presence of activated effectors in the peripheral blood and the decline following removal of the implant is compatible with the presumption that the T cells are activated in metallosis patients, but not in patients with aseptic loosening. Systemic activation of T cells could be a prerequisite for their emigration from the blood vessel into an inflammatory site. The perivascular accumulation of lymphocytes [20,21] as it is described in patients with metallosis as "ALVAL" (aseptic lymphocytic vasculitis associated lesion) could reflect this T cell trafficking.

How a systemic T cell activation occurs is still under investigation. A specific immune response to metal ions in terms of a type IV hypersensitivity is not regularly seen in metallosis patients [23,24,38]. Possibly, metal-loaded monocytes or macrophages emigrate to the lymph nodes encountering T cells there, and activate the cells by a not yet defined mechanism. Alternatively, metal ions in the blood could affect T cells and T cell function, as it has already been reported for tissue cells [39,40].

## Conclusion

In conclusion, we consider metallosis as a clinical entity, caused by a local inflammatory response, which according to histomorphology and the composition of the cellular infiltrate classifies as an acute phase of chronic inflammatory disease. The inflammation persists because of the constant triggering wear particles, which are released from the implant over time, and possibly also by self-perpetuating cytokine-driven processes. The proinflammatory environment favors the generation of bone resorbing cells; loss of bone ensues in implant loosening, which then causes the major symptoms of metallosis: pain and a reduced range of motion.

## Competing interests

The authors declare that they have no competing interests.

## Authors’ contributions

The following authors have made substantial contributions to conception and design of the study: UD, TG, GMH. -The following authors have made substantial contributions to acquisition of data or analysis and interpretation of data: UD, TG, GMH, FL, JR. -The following authors have been involved in drafting the manuscript or revising it critically for important intellectual content: UD, TG, GMH, FL, JR, JPK, BL, VE. -The following authors have given final approval of the version to be published and agree to be accountable for all aspects of the work in ensuring that questions related to the accuracy or integrity of any part of the work are appropriately investigated and resolved: UD, TG, GMH, FL, JR, JPK, BL, VE. All authors read and approved the final manuscript.

## References

[B1] Learmonth ID Young C Rorabeck C The operation of the century: total hip replacement Lancet 2007 370 9597 1508 1519 10.1016/S0140-6736(07)60457-7 17964352

[B2] Goodman SB Wear particles, periprosthetic osteolysis and the immune system Biomaterials 2007 28 34 5044 5048 10.1016/j.biomaterials.2007.06.035 17645943 PMC2065897

[B3] Shimmin A Beaule PE Campbell P Metal-on-metal hip resurfacing arthroplasty J Bone Joint Surg Am 2008 90 3 637 654 10.2106/JBJS.G.01012 18310716

[B4] Grigoris P Roberts P Panousis K Jin Z Hip resurfacing arthroplasty: the evolution of contemporary designs Proc Inst Mech Eng H 2006 220 2 95 105 16669379 10.1243/095441105X69042

[B5] Ollivere B Darrah C Barker T Nolan J Porteous MJ Early clinical failure of the Birmingham metal-on-metal hip resurfacing is associated with metallosis and soft-tissue necrosis J Bone Joint Surg (Br) 2009 91 8 1025 1030 19651828 10.1302/0301-620X.91B8.21701

[B6] de Steiger RN Hang JR Miller LN Graves SE Davidson DC Five-year results of the ASR XL acetabular system and the ASR hip resurfacing system: an analysis from the Australian Orthopaedic Association National Joint Replacement Registry J Bone Joint Surg Am 2011 93 24 2287 2293 22258775 10.2106/JBJS.J.01727

[B7] Steele GD Fehring TK Odum SM Dennos AC Nadaud MC Early failure of articular surface replacement XL total hip arthroplasty J Arthroplasty 2011 26 6 Suppl 14 18 21550764 10.1016/j.arth.2011.03.027

[B8] Langton DJ Jameson SS Joyce TJ Hallab NJ Natu S Nargol AV Early failure of metal-on-metal bearings in hip resurfacing and large-diameter total hip replacement: a consequence of excess wear J Bone Joint Surg (Br) 2010 92 1 38 46 20044676 10.1302/0301-620X.92B1.22770

[B9] Cuckler JM Metal-on-metal surface replacement: a triumph of hope over reason: affirms Orthopedics 2011 34 9 e439 e441 21902124 10.3928/01477447-20110714-21

[B10] MacDonald SJ Metal-on-metal total hip arthroplasty: the concerns Clin Orthop Relat Res 2004 429 86 93 15577471 10.1097/01.blo.0000150309.48474.8b

[B11] Park YS Moon YW Lim SJ Yang JM Ahn G Choi YL Early osteolysis following second-generation metal-on-metal hip replacement J Bone Joint Surg Am 2005 87 7 1515 1521 10.2106/JBJS.D.02641 15995119

[B12] Haddad FS Thakrar RR Hart AJ Skinner JA Nargol AV Nolan JF Gill HS Murray DW Blom AW Case CP Metal-on-metal bearings: the evidence so far J Bone Joint Surg (Br) 2011 93 5 572 579 21511920 10.1302/0301-620X.93B4.26429

[B13] Benson MK Goodwin PG Brostoff J Metal sensitivity in patients with joint replacement arthroplasties Br Med J 1975 4 5993 374 375 10.1136/bmj.4.5993.374 1192078 PMC1675237

[B14] Pazzaglia UE Ceciliani L Wilkinson MJ Dell'Orbo C Involvement of metal particles in loosening of metal-plastic total hip prostheses Arch Orthop Trauma Surg 1985 104 3 164 174 10.1007/BF00454694 3904669

[B15] Willert HG Semlitsch M Reactions of the articular capsule to wear products of artificial joint prostheses J Biomed Mater Res 1977 11 2 157 164 10.1002/jbm.820110202 140168

[B16] Hallab N Merritt K Jacobs JJ Metal sensitivity in patients with orthopaedic implants J Bone Joint Sur 2001 83 3 428 10.1302/0301-620X.83B3.9674 11263649

[B17] Willert HG Buchhorn GH Fayyazi A Flury R Windler M Koster G Lohmann CH Metal-on-metal bearings and hypersensitivity in patients with artificial hip joints, a clinical and histomorphological study J Bone Joint Surg Am 2005 87 1 28 36 10.2106/JBJS.A.02039pp 15637030

[B18] Huber M Reinisch G Trettenhahn G Zweymuller K Lintner F Presence of corrosion products and hypersensitivity-associated reactions in periprosthetic tissue after aseptic loosening of total hip replacements with metal bearing surfaces Acta Biomater 2009 5 1 172 180 10.1016/j.actbio.2008.07.032 18725188

[B19] Davies AP Willert HG Campbell PA Learmonth ID Case CP An unusual lymphocytic perivascular infiltration in tissues around contemporary metal-on-metal joint replacements J Bone Joint Surg Am 2005 87 1 18 27 10.2106/JBJS.C.00949 15634811

[B20] Natu S Sidaginamale RP Gandhi J Langton DJ Nargol AV Adverse reactions to metal debris: histopathological features of periprosthetic soft tissue reactions seen in association with failed metal on metal hip arthroplasties J Clin Pathol 2012 65 5 409 418 10.1136/jclinpath-2011-200398 22422805

[B21] Campbell P Ebramzadeh E Nelson S Takamura K De Smet K Amstutz HC Histological features of pseudotumor-like tissues from metal-on-metal hips Clin Orthop Relat Res 2010 468 9 2321 2327 10.1007/s11999-010-1372-y 20458645 PMC2914255

[B22] Ingham E Fisher J The role of macrophages in osteolysis of total joint replacement Biomaterials 2005 26 11 1271 1286 10.1016/j.biomaterials.2004.04.035 15475057

[B23] Hallab N Metal sensitivity in patients with orthopedic implants J Clin Rheumatol 2001 7 4 215 218 10.1097/00124743-200108000-00004 17039137

[B24] Kwon YM Thomas P Summer B Pandit H Taylor A Beard D Murray DW Gill HS Lymphocyte proliferation responses in patients with pseudotumors following metal-on-metal hip resurfacing arthroplasty J Orthop Res 2010 28 4 444 450 19834954 10.1002/jor.21015

[B25] Baldwin L Flanagan BF McLaughlin PJ Parkinson RW Hunt JA Williams DF A study of tissue interface membranes from revision accord knee arthroplasty: the role of T lymphocytes Biomaterials 2002 23 14 3007 3014 10.1016/S0142-9612(02)00059-5 12069343

[B26] Kotsougiani D Pioch M Prior B Heppert V Hansch GM Wagner C Activation of T lymphocytes in response to persistent bacterial infection: induction of CD11b and of toll-like receptors on T Cells Int J Inflamm 2010 2010 526740 10.4061/2010/526740 PMC2989653 21151520

[B27] Gaida MM Mayer B Stegmaier S Schirmacher P Wagner C Hänsch GM Polymorphonuclear neutrophils in osteomyelitis: link to osteoclast generation and bone resorption European J of Inflammation 2012 10 413 426

[B28] Pfeilschifter J Chenu C Bird A Mundy GR Roodman GD Interleukin-1 and tumor necrosis factor stimulate the formation of human osteoclastlike cells in vitro J Bone Miner Res 1989 4 1 113 118 2785743 10.1002/jbmr.5650040116

[B29] Ha J Lee Y Kim HH CXCL2 mediates lipopolysaccharide-induced osteoclastogenesis in RANKL-primed precursors Cytokine 2011 55 1 48 55 10.1016/j.cyto.2011.03.026 21507677

[B30] Henderson B Nair SP Hard labour: bacterial infection of the skeleton Trends Microbiol 2003 11 12 570 577 10.1016/j.tim.2003.10.005 14659689

[B31] Mandelin J Li TF Liljestrom M Kroon ME Hanemaaijer R Santavirta S Konttinen YT Imbalance of RANKL/RANK/OPG system in interface tissue in loosening of total hip replacement J Bone Joint Surg (Br) 2003 85 8 1196 1201 10.1302/0301-620X.85B8.13311 14653607

[B32] Wada T Nakashima T Hiroshi N Penninger JM RANKL-RANK signaling in osteoclastogenesis and bone disease Trends Mol Med 2006 12 1 17 25 10.1016/j.molmed.2005.11.007 16356770

[B33] Wagner C Kondella K Bernschneider T Heppert V Wentzensen A Hansch GM Post-traumatic osteomyelitis: analysis of inflammatory cells recruited into the site of infection Shock 2003 20 6 503 510 10.1097/01.shk.0000093542.78705.e3 14625473

[B34] Gesser B Lund M Lohse N Vestergaad C Matsushima K Sindet-Pedersen S Jensen SL Thestrup-Pedersen K Larsen CG IL-8 induces T cell chemotaxis, suppresses IL-4, and up-regulates IL-8 production by CD4+ T cells J Leukoc Biol 1996 59 3 407 411 8604020 10.1002/jlb.59.3.407

[B35] Taub DD Lloyd AR Wang JM Oppenheim JJ Kelvin DJ The effects of human recombinant MIP-1 alpha, MIP-1 beta, and RANTES on the chemotaxis and adhesion of T cell subsets Adv Exp Med Biol 1993 351 139 146 10.1007/978-1-4615-2952-1_15 7524282

[B36] Qin S LaRosa G Campbell JJ Smith-Heath H Kassam N Shi X Zeng L Buthcher EC Mackay CR Expression of monocyte chemoattractant protein-1 and interleukin-8 receptors on subsets of T cells: correlation with transendothelial chemotactic potential Eur J Immunol 1996 26 3 640 647 10.1002/eji.1830260320 8605932

[B37] Sallusto F Geginat J Lanzavecchia A Central memory and effector memory T cell subsets: function, generation, and maintenance Annu Rev Immunol 2004 22 745 763 10.1146/annurev.immunol.22.012703.104702 15032595

[B38] Innocenti M Carulli C Matassi F Carossino AM Brandi ML Civinini R Total knee arthroplasty in patients with hypersensitivity to metals Int Orthop 2014 38 2 329 333 10.1007/s00264-013-2229-2 24389947 PMC3923923

[B39] Hart AJ Hester T Sinclair K Powell JJ Goodship AE Pele L Fersht NL Skinner J The association between metal ions from hip resurfacing and reduced T-cell counts J Bone Joint Surg (Br) 2006 88 4 449 454 16567777 10.1302/0301-620X.88B4.17216

[B40] Wagner M Klein CL van Kooten TG Kirkpatrick CJ Mechanisms of cell activation by heavy metal ions J Biomed Mater Res 1998 42 3 443 452 10.1002/(SICI)1097-4636(19981205)42:3<443::AID-JBM14>3.0.CO;2-H 9788508

